# Mechanisms of 3-Hydroxyl 3-Methylglutaryl CoA Reductase in Alzheimer’s Disease

**DOI:** 10.3390/ijms25010170

**Published:** 2023-12-22

**Authors:** Xun Zhou, Xiaolang Wu, Rui Wang, Lu Han, Huilin Li, Wei Zhao

**Affiliations:** 1 Science and Technology Innovation Center, Guangzhou University of Chinese Medicine, Guangzhou 510405, China; 20201110948@stu.gzucm.edu.cn (X.Z.); 20211110926@stu.gzucm.edu.cn (X.W.); 20211110955@stu.gzucm.edu.cn (R.W.); lu20190606@126.com (L.H.); 2 Department of Endocrinology, The Fourth Clinical Medical College of Guangzhou University of Chinese Medicine, Shenzhen 518033, China; sztcmlhl@163.com

**Keywords:** Alzheimer’s disease, HMGCR, lipid mediators, lipid metabolism, neuroinflammation

## Abstract

Alzheimer’s disease (AD) is the most common neurodegenerative disease worldwide and has a high incidence in the elderly. Unfortunately, there is no effective therapy for AD owing to its complicated pathogenesis. However, the development of lipid-lowering anti-inflammatory drugs has heralded a new era in the treatment of Alzheimer’s disease. Several studies in recent years have shown that lipid metabolic dysregulation and neuroinflammation are associated with the pathogenesis of AD. 3-Hydroxyl 3-methylglutaryl CoA reductase (HMGCR) is a rate-limiting enzyme in cholesterol synthesis that plays a key role in cholesterol metabolism. HMGCR inhibitors, known as statins, have changed from being solely lipid-lowering agents to neuroprotective compounds because of their effects on lipid levels and inflammation. In this review, we first summarize the main regulatory mechanism of HMGCR affecting cholesterol biosynthesis. We also discuss the pathogenesis of AD induced by HMGCR, including disordered lipid metabolism, oxidative stress, inflammation, microglial proliferation, and amyloid-β (Aβ) deposition. Subsequently, we explain the possibility of HMGCR as a potential target for AD treatment. Statins-based AD treatment is an ascent field and currently quite controversial; therefore, we also elaborate on the current application prospects and limitations of statins in AD treatment.

## 1. Introduction

Alzheimer’s disease (AD) is defined as a degenerative neurological condition that mostly occurs later in life and causes memory and cognitive impairment. The development of AD symptoms consists of the following three stages: (1) the preclinical phase; (2) mild cognitive impairment (MCI); and (3) dementia. Of these, the MCI stage is an intermediate transitional period between normal older adulthood and AD. It has been estimated that nearly 40 to 60% of patients with amnestic MCI will develop full-blown AD dementia [1]. As the global population ages, it is becoming increasingly important to address the social and economic challenges associated with AD [2]. However, there is no cure for AD, and treatment appears to be more effective in the early stages, so developing a treatment for MCI has recently become a research focus. The early stages of AD are linked to multiple pathological mechanisms and risk factors, ranging from neuroinflammation and neuronopathies to disordered lipid metabolism [1,3]; therefore, memory can be improved at the early stages by lowering lipid levels and inflammation.

Cholesterol homeostasis is maintained through the complex regulation of the synthesis, transport, and elimination of excess cholesterol in the brain. Dysfunctional lipid metabolism, especially cholesterol metabolism, is particularly important in neurodegenerative diseases since brain sterol synthesis is critical for synaptic function and the regulation of various functions of neuronal metabolism [4]. Of these, the synthesis of brain cholesterol primarily occurs via two processes: 3-hydroxyl 3-methylglutaryl CoA reductase (HMGCR) synthesis and APOE/low-density lipoprotein (LDL) receptor cascade lipoprotein internalization [5,6]. HMGCR is one of the most important coregulators of cholesterol synthesis and can activate cholesterol production in mammalian cells. In contrast, apolipoprotein E (ApoE) plays a role in the recycling and cell-to-cell transport of cerebral cholesterol [7]. To maintain cholesterol homeostasis, cholesterol synthesized by HMGCR is converted to 24(S)-hydroxyl cholesterol (24S-OHC) via cytochrome P450 46A1 (CYP46A1), which readily crosses the blood–brain barrier (BBB) and enters peripheral circulation. It is then absorbed by plasma lipoproteins and transported to the liver for metabolism [8]. Accordingly, inhibiting HMGCR activity is a new and promising way to lower cholesterol levels. Experimental studies have shown that HMGCR can induce proinflammatory responses in obese mice that were caused by a high-fat diet [9]. Interestingly, abnormalities in lipid metabolism and inflammation are both major risk factors for AD. For example, mice fed with a high-fat diet can experience increased amyloid-β (Aβ) accumulation and accelerated development of cognitive impairment [10,11]. Notably, the excessive deposition of Aβ can affect cholesterol metabolism in turn by increasing the levels of the HMGCR, APOE, and ATP-binding cassette transporter A1 (ABCA1) proteins [12]. Furthermore, lipid peroxidation and lipoprotein imbalance lead to an increase in the amounts of inflammatory factors in the arterial endothelium in the early stages of Alzheimer’s disease, resulting in endothelial dysfunction. Endothelial dysfunction leads to dermal lipoprotein accumulation and leukocyte adhesion [13], which promotes cholesterol accumulation and cellular waste deposition, ultimately leading to chronic inflammation [14]. Specifically, inflammation of cerebral vascular endothelial cells is an important process in the neurodegeneration observed in AD patients. Hence, Alzheimer’s disease is generally regarded as a chronic disease induced by the interaction of lipids with inflammatory responses. In light of the treatment of hypercholesterolemia for neuroprotection in the early stages of AD [15,16], in recent years, experts have focused on drugs or treatments that can lower cholesterol and alleviate neuroinflammation to prevent the onset of cognitive disorders. In this paper, we propose a hypothesis that HMGCR stimulates an increase in oxidized sterol toxicity through lipid disorders and inflammatory responses, leading to neuronal damage in the brain, which in turn serves as a novel target for the treatment of AD.

HMGCR inhibitors (statins) are the most common lipid-lowering agents for the treatment of dyslipidemia [17]. There are currently six different types of statins that have been approved by the Food and Drug Administration for clinical use. In general, statins are not currently used directly in the treatment of AD. However, these drugs have been proposed as candidate treatments for Alzheimer’s disease due to their pleiotropic effects, including cholesterol reduction, endothelial protection, and antioxidant and anti-inflammatory properties, all of which are risk factors for AD. Therefore, we also discuss the possibility of shifting statins from a reliable drug in the clinical setting for dyslipidemia to a candidate for the prevention and treatment of AD.

In summary, since some recent reviews have comprehensively summarized the latest progress in respect of the molecular mechanism of HMGCR, we focus here on the current understanding of the pathogenesis of AD induced by HMGCR and the AD defense mechanisms exhibited by HMGCR inhibitors. We also present the promise and limitations of statins for the treatment of neurodegenerative diseases in response to the conflict and controversy over the potential role of statins in AD neuropathology.

## 2. HMGCR-Induced Cholesterol Synthesis Mechanism

**Figure 1:**
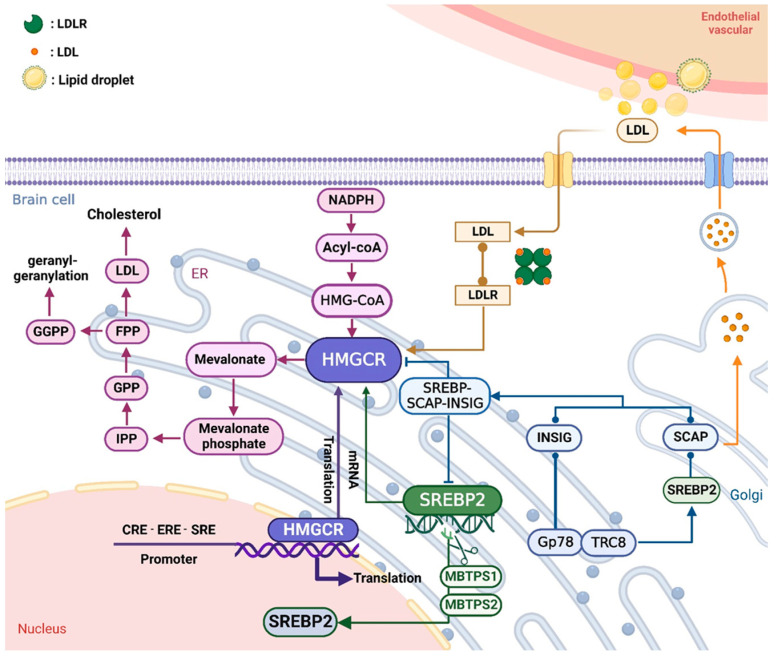
HMGCR catalyzes the NADPH-dependent conversion of HMG-CoA to mevalonate. Mevalonate is phosphorylated by mevalonate kinase and then metabolized to isopentenyl pyrophosphate (IPP), which promotes the formation of acetylene pyrophosphate (FPP) and geranylgeranyl pyrophosphate (GGPP) from IPP through the mevalonate pathway. In addition, LDLR can promote plasma LDL catabolism and reduce plasma cholesterol concentration by inhibiting HMGCR activity. SREBP cleavage activator protein (SCAP) can bind to *INSIG1* and *INSIG2* to form the SREBP/SCAP/INSIG complex, which is transmitted to the Golgi apparatus if necessary. Among them, sterol regulatory element binding protein-2 (SREBP-2) is retained as a membrane-bound precursor of the endoplasmic reticulum (ER) and binds to SCAP to sense cholesterol. Next, SREBP-2 is sequentially cleaved by membrane-bound transcription factor site-1 protease (MBTPS1) and MBTPS2 and transported to the nucleus. Interestingly, the SREBP/SCAP complex is retained in the endoplasmic reticulum at higher sterol concentrations, inhibiting SREBP-mediated transcription and HMGCR production. INSIGs can also link HMGCR to the E3 ligases gp78 and TRC8 to participate in the ubiquitination of HMGCR. HMGCR promoter regions such as sterol regulatory element (SRE), cyclic AMP response element (CRE), and estrogen response element (ERE) can activate HMGCR transcriptional activity. Created from https://app.biorender.com (accessed on 28 November 2022).

In the human body, cholesterol is synthesized mainly from mevalonate, which is the most crucial part of the first step of cholesterol synthesis. The mevalonate pathway acts on endogenous and exogenous cholesterol uptake and production, cholesterol conversion to bile acids and steroids, and lipid balance. It has been found that the pre-cholesterol gene *HMGCR* is involved in the cholesterol synthesis pathway. HMGCR catalyzed by acetyl-CoA is a membrane-bound glycoprotein consisting of 888 amino acids and it acts on the mevalonate pathway, which is the initial control point for endogenous cholesterol biosynthesis. HMGCR activity and quantity are regulated by cholesterol biosynthesis through several mechanisms, including negative feedback regulation mechanisms derived from mevalonate-induced sterol and nonsterol metabolite [18], *HMGCR* mRNA synthesis [19], HMGCR protein degradation [20], hormonal regulation [21], and gene regulation and genetic diversity [22]. These mechanisms interact to regulate cholesterol homeostasis. In addition, HMGCR is the main rate-limiting enzyme in negative feedback regulation, and its concentration directly affects cholesterol synthesis (Figure 1).

### 2.1. Mevalonate Pathway

Cholesterol is an essential lipid for cell function and membrane integrity. Balanced cholesterol metabolism is essential for human functioning. Therefore, the cellular level and distribution of cholesterol must be strictly regulated [23]. The mevalonate pathway is the main pathway through which cells acquire cholesterol. This pathway involves more than 20 enzymes, among which HMGCR is a key rate-limiting enzyme in cholesterol production. HMGCR catalyzes the NADPH-dependent conversion of HMG-CoA to mevalonate, which is the first step in cholesterol production [24]. Mevalonate is phosphorylated by mevalonate kinase and then metabolized to isopentenyl pyrophosphate (IPP) [25,26], which promotes the formation of acetylene pyrophosphate (FPP) and geranylgeranyl pyrophosphate (GGPP) from IPP through the mevalonate pathway. As a precursor of cholesterol and a key mevalonate pathway product, FPP can further promote the production of lipid products, such as polyphenols and ubiquitin ketones, which affect the oxidative stress response in vivo [27]. Of course, the transcription and translation mechanism of HMGCR can also directly regulate the mevalonate pathway [28]. In addition, both oxysterols and mevalonate pathway products can accelerate HMGCR degradation, which is a classic example of metabolically controlled feedback regulation [29,30].

Other evidence suggests that HMGCR activity increases with LDL starvation and is strongly inhibited with LDL hypersaturation. Although HMGCR mainly aggregates in liver cells, it is also widely distributed in the brain [31]. Brain sterols are also metabolized by the low-density lipoprotein receptor (LDLR) pathway and distributed in the blood by lipoproteins. In this pathway, LDL-C binds to LDL-C particles, LDL-C endocytosis occurs, and LDL-C particles are absorbed and eliminated, ultimately reducing blood cholesterol levels [32,33]. However, the reduction in LDLR can stimulate HMGCR to increase cholesterol synthesis in vivo. Conversely, when sufficient cholesterol is present in the cells, HMGCR undergoes proteolysis, thereby reducing its amount. Since HMGCR is the rate-limiting enzyme for cholesterol biosynthesis, it can directly or indirectly sense intracellular cholesterol levels and inhibit cholesterol synthesis. Thus, as the key point of cholesterol metabolism regulation, HMGCR is considered one of the major targets for the management of dyslipidemia [34]. It has been reported that cholesterol biosynthesis is regulated strictly by the sterol-induced degradation of HMGCR and sterol-regulated element-binding proteins, which regulate the transcription of all cholesterol-producing genes to prevent excessive cholesterol from becoming toxic [35]. On the one hand, cholesterol silences the HMGCR gene through a negative feedback mechanism. Furthermore, LDLR can promote plasma LDL catabolism and reduce plasma cholesterol concentration by inhibiting HMGCR activity [18]. Cholesterol also expedites HMGCR degradation by promoting HMGCR ubiquitination [36]. Both of these mechanisms complement each other and ultimately lead to a reduction in HMGCR concentration and cholesterol production, thereby lowering cholesterol levels.

### 2.2. SREBP-2 Transcriptional Regulation

Studies have revealed that nuclear transcription factors family SREBPs, SREBP cleavage activator protein (SCAP), and insulin-inducible genes (*INSIG1* and *INSIG2*) are all involved in the regulation of cholesterol synthesis [37,38]. SREBP-1 mainly promotes the formation of fatty acids and triglycerides, while SREBP-2 preferentially controls cholesterol synthesis. SREBP-2 is retained as a membrane-bound precursor of the endoplasmic reticulum (ER) and binds to SREBP cleavage activator protein (SCAP) to sense cholesterol [39,40]. SCAP can bind to *INSIG1* and *INSIG2* to form the SREBP/SCAP/ INSIG complex, which is transmitted to the Golgi apparatus if necessary. Next, SREBP-2 is sequentially cleaved by MBTPS1 and MBTPS2 and transported to the nucleus [41,42], where SREBP induces the expression of HMGCR. Interestingly, the SREBP/SCAP complex is retained in the endoplasmic reticulum at higher sterol concentrations, inhibiting SREBP-mediated transcription and HMGCR production. Furthermore, INSIGs are essential for regulating SREBP-dependent transcription and HMGCR degradation. INSIGs can also link HMGCR to the E3 ligases gp78 and TRC8 to participate in the ubiquitination of HMGCR [38,43,44], thereby promoting its degradation to reduce the production of cholesterol. In addition, the ligase gp78, an endoplasmic reticulum-enclosed protein, binds to ER-resident proteins INSIG-1 and INSIG-2, promoting HMGCR degradation when sterols are consumed [45,46]. One study showed that the specific knockdown of GP78 reduces SREBP levels, resulting in an increase in INSIGs and a decrease in cholesterol synthesis [47]. Hence, Gp78 deletion increases the foundation level of HMGCR protein, and sterol-induced degradation is seriously diminished [30]. On the one hand, lessening the production of cholesterol triggers the SREBP pathway, which increases the transcription of the HMGCR gene [48]. Additionally, cholesterol can maintain HMGCR protein stability by blocking HMGCR ubiquitination induced by hormonal regulation, such as INSIG-mediated HMGCR degradation [49,50]. Along with a sterol regulatory element (SRE), the HMGCR promoter region also contains a cyclic AMP response element (CRE) and an estrogen response element (ERE), which activate HMGCR transcriptional activity [47]. Estrogen activates the HMGCR signaling pathway by binding to ERE and promotes an increase in total cholesterol and triglyceride levels [51]. Cholesterol is essential for ovarian steroidogenesis, while reducing the level of estradiol can inhibit the expression of HMGCR. Some researchers have revealed that gene polymorphisms of HMGCR are related to its function and phenotype. For example, neurodegenerative disease is more likely to develop when someone carries the variant *HMGCR* alleles [52].

## 3. HMGCR-Mediated Neuroinflammation

**Figure 2:**
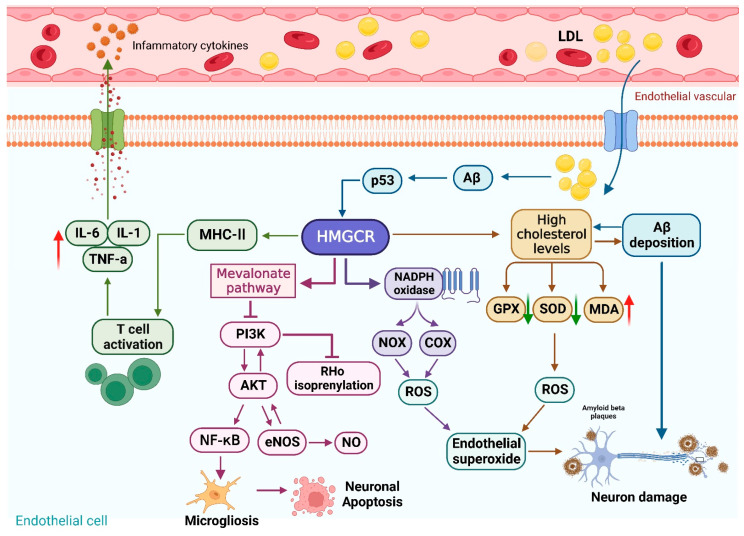
HMGCR-mediated inflammatory response is mainly caused by oxidative stress and neuroinflammatory mediators, microglial proliferation, and Aβ deposition. HMGCR upregulates MHC-II expression to activate T-cell activation, thereby increasing the release of the proinflammatory cytokines Interleukin-1 (IL-1) and Interleukin-6 (IL-6) and the synthesis of tumor necrosis factor (TNF-α). High cholesterol levels drastically decrease the activity of brain antioxidant–detoxifying enzymes, including glutathione peroxidase (GPX) and superoxide dismutase (SOD), while increasing the production of malondialdehyde (MDA). Arrows red (stimulate/increase), green arrows (inhibit/decrease). Created from https://app.biorender.com (accessed on 28 November 2022).

Dysregulation of lipid metabolism in the peripheral circulation can activate inflammatory factors [53,54] and then induce central inflammation [55,56]. In the previous section, we discussed the cholesterol synthesis metabolism caused by HMGCR. Next, we will review the pathological responses of HMGCR-induced neuroinflammation. There is significant proof that the direct direction of cholesterol-oxygenated products, also known as sterols, increases the expression of many inflammatory cytokines and chemokines in the brain; this increase is a neuropathological factor. The activation of glial cells, the production of many chemokines, and the release of neurotoxic substances occur concurrently with neuroinflammation. In addition, activated glial proliferation is accompanied by an increase in inflammatory mediators, including tumor necrosis factor (TNF-α) and (Interleukin-6) IL-6. The activation of HMGCR can increase brain cholesterol levels and induce oxidative stress, neuroinflammatory mediators, microglial proliferation, Aβ deposition, and apoptosis, leading to cognitive dysfunction (Figure 2).

### 3.1. Oxidative Stress

Oxidative stress refers to cellular oxidative damage caused by the unregulated oxidation of lipids, proteins, and nucleic acids by reactive oxygen species (ROS), which leads to antioxidant defense. With excessive oxidative stress, oxidative sterols accumulate due to the disordered oxidation of cholesterol by ROS. Metabolic derangements stemming from excessive cholesterol oxidation may be one of the mechanistic pathways leading to neuronal damage and cognitive dysfunction. HMGCR inhibits superoxide dismutase 3 (SOD3) and glutathione (GSH) through the PI3K/Akt pathway [57] and promotes Rho-isopentenylation [58], resulting in a severe oxidative stress response. As mentioned above, HMGCR can cause metabolic disorders and raise cholesterol levels. High cholesterol levels drastically decrease the activity of brain antioxidant–detoxifying enzymes, including glutathione peroxidase (GPX) and superoxide dismutase (SOD), while increasing the production of malondialdehyde (MDA), a particular biomarker of lipid peroxidation [59]. Indeed, the brain’s high oxygen consumption, higher neuronal metabolic rates, and lower antioxidant defense all make the brain more vulnerable to oxidative stress.

HMGCR can form endothelial superoxide generated by NADPH oxidases (NOXs) and cyclooxygenase (COX)-mediated ROS generation, which ultimately leads to cerebral infarction [60,61]. Conversely, the inhibition of HMGCR can increase the level of nitric oxide synthase to repair endothelial cell damage while alleviating oxidative stress damage to the nervous system. The inhibition of HMGCR can activate the angiotensin AT-1 receptor and significantly reduce lipid peroxidation and ROS levels, which suggests that HMGCR inhibition ameliorates endothelial damage and restores the blood–brain barrier (BBB) [62]. In contrast, Zhang Y et al. [63] found that the upregulation of *HMGCR* mRNA abundance greatly increases liver lipid levels but significantly decreases the NO and inducible nitric oxide synthase (iNOS) level while promoting oxidative stress.

Oxidative stress is associated with the overproduction of ROS. Nevertheless, increased intracellular ROS caused by high oxysterol may alter cellular protein levels, trigger neuronal apoptosis, and activate inflammatory pathways in neurons [64]. Oxidative stress is an early event in the pathogenesis of AD [65]. It induces the activation of astrocytes and microglia, followed by the release of proinflammatory molecules, which in turn release toxic free radicals, exacerbating neuronal damage [66]. In the AD brain, oxidative sterols accumulate continuously and produce cytotoxicity, mainly due to the disruption of BBB integrity [67], leading to neurodegeneration by enhancing oxidative stress and inflammation. In conclusion, as a product of cholesterol oxidation, oxidative stress can affect the production of cholesterol and oxysterols and play a significant role in regulating cholesterol homeostasis in the central nervous system (CNS).

### 3.2. Inflammation

In addition to oxidative stress, HMGCR is associated with neuroinflammation. The inhibition of HMGCR, the rate-limiting enzyme in the mevalonate (MVA) biosynthesis pathway and its downstream isoprene compounds, reduced isoprene and affected cell proliferation and the inflammatory response. Moreover, HMGCR inhibits the PI3K/Akt pathway and attenuates the expression of eNOS and nuclear factor-kappa B (NF-κB) in the mevalonate pathway [68,69]. The signaling pathway activated by eNOS is thought to be a key player in this process. Interestingly, eNOS can activate the PI3K/Akt pathway in turn and inhibit Rho isoprenylation. Lipid modification of Rho proteins such as RhoA and Cdc42 activates microgliosis and the inflammatory signaling cascade [70,71] and participates in the signaling pathways required for NF-κB activity and helps regulate the transcription of proinflammatory genes. Of Note, the isoprenylation of Rho protein has emerged as a key target of neuroprotective medicines, particularly statins, in illnesses caused by neuroinflammation [72].

On the one hand, HMGCR is known to be a proinflammatory compound that upregulates MHC-II expression to activate T-cell activation, thereby increasing the release of the proinflammatory cytokines Interleukin-1 (IL-1) and IL-6 and the synthesis of TNF-α [73,74]. In addition, HMGCR correspondingly increases the levels of proinflammatory mediators and leukocyte adhesion molecules through the upregulation of Toll-like receptors. However, the inhibition of HMGCR can upregulate the levels of nitric oxide synthase to repair endothelial cell damage, while relieving oxidative stress damage to the nervous system. On the other hand, HGMCR can stimulate synaptic structural changes in neurons and synapses after brain damage while restraining the release and expression of BDNF.

### 3.3. Microgliosis

It has been reported that neurodegenerative diseases induced by neuroinflammation are primarily caused by the diminished cholinergic control of brain microglia [75]. Glial hyperplasia leads to cortical and hippocampal or basal ganglia damage in neurodegenerative diseases. In addition, gliosis is the basis of neuronal destruction and can induce the destruction of physiological structures associated with AD [76]. On the one hand, glial cell proliferation can be mediated by increased levels of inflammatory-related factors, such as proinflammatory cytokines, iNOS, and COXs [77]. Systemic inflammation promotes the activation of microglia to generate neurotoxic substances, such as ROS and reactive nitrogen species (RNS), which damage healthy neurons [78,79].

On the other hand, HMGCR can stimulate the nuclear factor kappa B (NF-κB) signaling pathway, which causes microglia to proliferate and promotes synaptic damage and neuronal apoptosis [80]. Microglia are the primary source of inflammatory factors in the brain. The NF-κB pathway can be blocked by inhibiting HMCGR, which subsequently reduces the activation of microglia and the expression of proinflammatory cytokines in brain tissue [81]. Together, HMGCR can not only stimulate inflammatory factors and microgliosis but also mediate the upregulation of iNOS and then stimulate the overproduction of NO, thus affecting the occurrence and development of AD [82].

### 3.4. Aβ Deposition

Aβ toxicity, insulin resistance, neuroinflammation, and the dysregulation of lipid homeostasis are hallmarks of Alzheimer’s disease. Aβ plaques have been shown to stimulate microgliosis and accelerate proinflammatory pathways activation, leading to oxidative stress and brain microstructure injury in AD patients [83]. Cholesterol accumulation and oxidative stress can cause the excessive deposition of Aβ, which mediates neurodegeneration and cognitive deficits in the brain [84]. HMGCR promotes the synthesis of cholesterol in the brain and increases the level of Aβ. Experimental studies have shown that HMGCR can affect the plasma cholesterol level by increasing the Aβ content and the microglial toxicity of Aβ. The vascular mechanism is the most obvious link between plasma cholesterol and AD. Vascular dysfunction promotes amyloid precursor protein (APP) synthesis and Aβ cleavage, which subsequently compromise vascular function. These processes form a vicious cycle and reinforce each other, thereby worsening vascular dysfunction and eventually leading to dementia. In addition, elevated LDL has been associated with increased Aβ42 and plasma APOB in AD patients in crossover studies [85]. The above findings imply that HMGCR may be a key factor in the onset of Alzheimer’s disease by affecting the level of Aβ.

On the one hand, Aβ(1–42) can increase cholesterol accumulation in the brain. Saeed K et al. [84] found that the injection of Aβ(1–42) can cause oxidative stress, neurological alterations, and cholesterol metabolism disorders. Owing to the high expression of p53 and HMGCR, amyloidosis was accelerated in Aβ(1–42)-treated animals, and cholesterol retention in the brain was enhanced. It was also demonstrated that Aβ(1–42) increases brain cholesterol levels via the p53/HMGCR axis to induce cognitive impairment. Additionally, Najem D et al. [86] found that nerve cells treated with Aβ42 had higher levels of HMGCR protein and c-Jun phosphorylation. This evidence indicates that Aβ42 may activate HMGCR, which then disrupts the balance of cholesterol and results in increased cholesterol accumulation in brain cells. On the other hand, an imbalance in cholesterol metabolism promotes the amyloid processing of Aβ peptides sheared by γ-secretase. Moreover, Aβ not only increases the ABCA1 protein level and significantly reduces cholesterol efflux but also directly affects HMGCR activity and the production of cholesterol in nerve cells. Zubillaga M et al. [87] obtained the same results in another neuronal cell line, i.e., the neuroblastoma cell line (SH-SY5Y). According to their studies, activating HMGCR, increasing ROS, and promoting Chol synthesis may promote APP redistribution in rafts, which contribute to amyloid processing of the protein, and then increase the level of Aβ.

## 4. HMGCR as a Therapeutic Target for AD

Brain cholesterol is essential for neuroregulatory proteins to be able to regulate glial cell development and myelin formation. In the central nervous system, cholesterol is produced primarily through the following two distinct but connected processes: HMGCR synthesis and the APOE/LDL receptor (LDLR) cascade. Remarkably, alterations in brain structure are restricted to AD neuropathological regions and occur before the clinical onset of AD [88]. Regarding the three clinical phases (NC, MCI, and AD), HMGCR mainly acts during the MCI stage, where it may also impact brain glucose metabolism [89,90] and contribute to the pathophysiological transition from MCI to AD [90,91]. When the rat hypothalamus is injected with HMGCR inhibitors, this not only alters insulin signaling and lipid storage metabolism via mevalonate channels but also increases the neuronal activity of the arcuate nucleus and the paraventricular nucleus (PVN) [92].

Interestingly, HMGCR shares a genetic relationship with APOE, which is the most significant cholesterol transporter in the brain. APOE4 is a risk factor for the early stages of AD, while both APOE2 mutation and G-negative polymorphism of HMGCR delay AD onset. Moreover, lipid neurobiologists consider APOE to be a crucial extracellular lipid transporter, while HMGCR is located in the ER and is essentially an intracellular organelle-specific protein that controls intracellular lipid synthesis [93]. The APOE/LDL pathway is mostly used in peripheral tissues to metabolize blood cholesterol [94]. Inhibiting HMGCR signaling enables LDLR on the cell membrane to bind to plasma LDL-C particles, thereby transporting hepatic cholesterol and allowing LDL-C to be absorbed and cleared, and vice versa. HMGCR may act on the signaling pathway of peripheral lipid metabolism through the APOE/LDL-mediated endocytic clearance pathway.

Heida A et al. fed mice a carbohydrate-rich diet and then found that HMGCR expression was upregulated, leading to increased hepatic and plasma cholesterol levels [95]. To further validate this finding, the researchers performed a reverse test and found that inhibiting the enzymatic activity of HMGCR can impair the isoprenylation of adipocyte proteins to prevent the browning of adipocytes, thereby lowering cholesterol level [96,97]. They then conducted a Mendelian randomization study on the human *HMGCR* gene and discovered that variation in the *HMGCR* gene coding region, which is connected to the regulation of LDL-C, impacts the patient’s risk for AD. The level of HMGCR in AD patients is positively correlated with AD-related cognitive impairment and brain microstructure damage, and variation in the *APOE* gene further increases the expression of HMGCR [98]. These changes may indicate that HMGCR plays a key role in brain lipid metabolism dysfunction and AD pathology. Overall, we believe that the neurodegeneration in AD may be the result of the imbalance in lipid metabolism caused by HMGCR.

**Figure 3:**
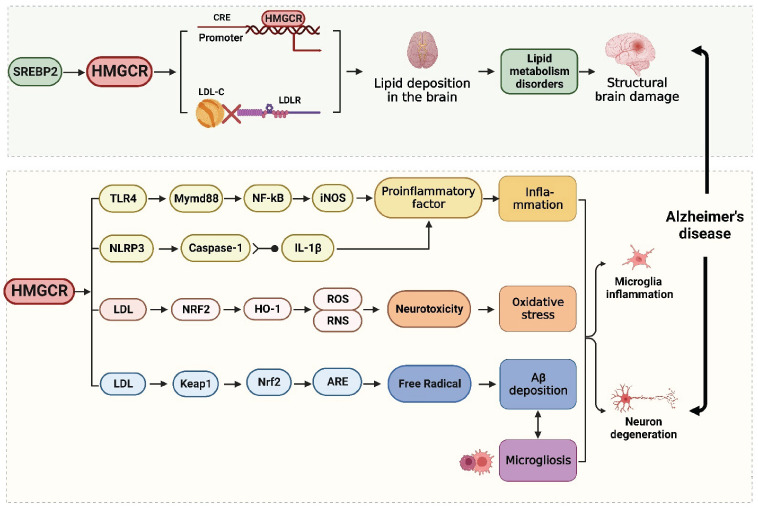
Cognitive dysfunction can result from activation of the HMGCR, which can also cause lipid metabolism disorder, oxidative stress, the release of neuroinflammatory mediators, and proliferation, Aβ deposition, and microglial proliferation. Red cross indicates separation of LDLR from plasma LDL-C particles. Created from https://app.biorender.com (accessed on 28 November 2022).

According to previous reports, the widespread consensus is that Alzheimer’s disease is a lipid-induced inflammatory disease. Maintaining cholesterol balance in the brain requires the strict regulation of cholesterol biosynthesis and oxidative sterol metabolism in the brain and circulation [99]. As stated above, HMGCR can not only cause disordered lipid metabolism but also induce oxidative stress, neuroinflammation, Aβ deposition, and microglial proliferation. First, the increased expression of HMGCR can promote the phosphorylation of IRS-1 and insulin resistance and stimulate nuclear factor-kB (NF-κB) through the TLR4/MyD88/NF-κB signaling cascade [100], which increases iNOS production to maintain the inflammatory response [101]. In particular, the NLRP3 inflammasome is a key factor in inflammation. After activating the NLRP3 sensor, HMGCR stimulates caspase-1 and IL-1β to be released into the systemic blood system through exocytosis or the cell membrane [102], causing inflammatory reactions and pathological effects. Second, activation of the HMGCR axis enhances the accumulation of amyloid and brain cholesterol, stimulates oxidative stress through the NRF2/HO-1 pathway, ROS, and RNS, and causes neuronal toxicity [103], which directly results in neuronal damage. These pathways allow inflammatory factors to move into the blood and quickly pass through the BBB, causing inflammatory reactions in neurons, damaging brain function and structure, and inducing abnormalities in the central nervous system [104]. Third, brain inflammation, a key neuropathological factor in the pathogenesis of AD, can directly induce the generation of cholesterol oxidation products and stimulate the expression of cytokines and chemokines [66]. Remarkably, the link between cholesterol and AD is supported by evidence that low cholesterol levels increase the production of soluble APP [105,106], while elevated cholesterol levels upregulate the formation of Aβ42, leading to the hyperphosphorylation of tau [107]. For instance, the activation of HMGCR increases the level of cholesterol that is synthesized, which leads to abnormal manifestations of the Keap1/Nrf2/ARE pathway [108,109], and the further induction of free radicals and oxidative stress, leading to Aβ deposition [110]. In addition, Aβ plaques have been shown to stimulate microgliosis and accelerate proinflammatory pathways, leading to oxidative stress and endothelial dysfunction and eventually causing brain cell structure abnormalities and leading to AD. Together, as a potential target for the treatment of AD, HMGCR may influence the development of AD by mediating the interaction between disordered lipid metabolism and inflammatory responses (Figure 3).

## 5. Potential and Controversy of Statins in AD Treatment

Statins, known as HMGCR inhibitors, are potent cholesterol-lowering medications for hypercholesterolemia treatment in clinical practice. Several statins can cross the BBB with the preservation of brain white matter through both peripheral and central effects, where they not only upregulate brain cholesterol recycling but also have pleotropic effects [111]. Based on evidence that HMGCR dysfunction is significantly associated with brain β-amyloid production and neuronal cell death, both of which are characteristic of AD, laboratory studies suggest that statins can reduce the production of Aβ [112]. Moreover, statins are proposed as crucial tools for the interventional treatment of AD by reducing the formation of Aβ [113,114] and inhibiting p-tau levels by interacting with microtubules [115]. Notably, statins have also been found to activate the expression of neurotrophic factors in brain cells and therefore reduce the risk of AD [116]. These results support the possibility that statins directly modulate cholesterol homeostasis as well as Aβ degradation in the CNS and suppress CNS inflammation to mitigate the effects of AD. Statins can also reduce the levels of inflammatory biomarkers such as transforming growth factor (TGF-β), C-reactive protein (CRP), IL-6, and NF-κB, for which statins are also recognized to be anti-inflammatory.

These results support the possibility that statins directly modulate cholesterol homeostasis in the CNS, reduce vascular risk factors, and suppress CNS inflammation to mitigate the effects of AD. Consequently, these properties increase the usefulness of statins, taking them from being simple lipid-lowering agents to being neuroprotective compounds. Different studies including preclinical to clinical studies have shown that statins have a neuroprotective effect against the development and progression of AD or its related pathological components (Table 1).

There is still controversy about whether statins can slow the progression of AD. Some experts think that statins may cross the BBB and modulate brain cholesterol metabolism, directly altering neurotransmission and synaptic plasticity [132]. Particularly, the cognitive effects mediated by statins may simply be related to their ability to lower blood cholesterol. The predictable effects of aggressive LDL-C-lowering therapy may explain the different clinical outcomes that have been associated with statin therapy, specifically when compared to the potential direct effects of statins on Aβ or other pathophysiological mechanisms in AD. However, other experts believe that, due to the pleiotropic nature of statins, they have failed to demonstrate any additional positive effect of statins on the cognitive function of patients with dementia and Alzheimer’s disease. Other clinical trials have reported no therapeutic benefit or even acute cognitive impairment in patients receiving this therapy [133,134].

One possible explanation is that statins lower cholesteryl esters and reduce the levels of the disease-causing proteins tau and Aβ produced by human neurons, but even at low concentrations, statins are toxic to astrocytes. This result explains the conflicting results of studies on statin therapy for AD. Theoretically, statins can lower tau levels in the body; however, the underlying mechanism and whether this effect extends to human neurons remain unclear.

Another possible explanation for the conflicting results is the progression of AD and the magnitude of cholesterol reduction among participants. One study suggests that [135] there was no benefit or harm in patients with mild-to-moderate AD who received statins compared with those who received placebo; however, the study only assessed the effects of the drug in individuals with normal cholesterol levels. This selection of patients is in stark contrast to a previous study [136] that assessed only subjects with high cholesterol levels and found that statins improved the treatment of AD patients. These conflicting results may be due to the cholesterol levels in the treatment population. It is reasonable to assume that statin therapy may be beneficial in AD, but only in those with high cholesterol levels, so the fact that statin therapy has little effect on cognitive function in AD patients with normal cholesterol levels is not surprising. In addition, the number of research objects, relatively short follow-up times, poor control of risk factors, and the susceptibility of observational studies to bias and confounding known and unknown factors may have limited the conclusions.

Collectively, there are several limitations to the current understanding of statin therapy for dementia. Heterogeneity in the diagnostic criteria for dementia, different subjects, inadequate duration of therapy or intervention, risk factors, and different methods of assessing statin use in the included studies may limit the generality of the results. Given the paucity of evidence for statins treatment in dementia, future randomized trials with standardized diagnostic approaches are needed to replicate these findings and determine whether statins have an effect on delaying or preventing AD.

## 6. Conclusions and Perspectives

As a multi-cause and multi-factor neurological disease, AD continues to be a top priority since no commercially available medication can cure it. Fortunately, experimental studies based on multiple therapeutic targets and approaches offer optimism for the eventual clinical conversion of anti-AD drugs at present. HMGCR-based anti-AD drugs can provide lipid-lowering-targeted and anti-inflammatory effects and have thus been receiving extensive attention in recent years. As a highly functional gene of AD, HMGCR provides multi-target effects while inducing lipid accumulation, oxidative stress, Aβ deposition, and microgliosis, which further aggravate damage to the central nervous system. Although the precise function of HMGCR-induced AD and the pathogenic mechanism involved in neuroinflammation have been explored, more mechanistic and functional research is required to demonstrate the significance of lipid metabolism in the HMGCR-AD pathogenesis axis, and particularly to determine whether and how dysfunctional cholesterol metabolism in the brain affects astrocyte reactivity.

In addition, although statins were previously considered a key drug for hypercholesterolemia, the failure of some clinical trials and their strong side effects when used for AD have cast doubt on their suitability. This may be related to the fact that statins reduce the biosynthesis of cholesterol in muscle cells, resulting in reduced cholesterol content in the plasma membrane, further leading to membrane instability and cell damage. At present, whether statins (HMGCR inhibitors) play a role in the early stages of AD and their specific mechanisms still needs to be clinically verified in a large population. However, AD-targeted strategies have not been abandoned completely. Innovations in drug design and development could allow statins to be used in combination with other molecules, or statins could be loaded into nanoparticles to combat side effects. In summary, HMGCR provides a novel therapeutic pathway and more appropriate strategies for AD treatment. Future studies on this significant neurodegenerative disease will be aided by the clinical development of more accurate and multi-targeted medications, which will usher in a new era of AD therapy.

## Figures and Tables

**Table 1:** Different studies of statins in AD treatment.

Study Type	Findings	Ref.
A preclinical study	Statins provide a neuroprotective effect through ameliorating memory deficits and inflammation in a mouse model of AD.	Atef et al. [117]
A preclinical study	Lipid-core nanocapsules containing statin ameliorate hypercholesterolemia induced cognitive impairment in adult rats.	Lorenzoni et al. [118]
A preclinical study	Statins modulate nicotinic acetylcholine receptor levels in rat hippocampal neurons in α7 and α4 neurons.	Borroni et al. [119]
A preclinical study	Statins rescue memory and granule cell maturation in a mouse model of AD.	Tong et al. [120]
A preclinical study	Statins effectively attenuate dyslipidemia-induced neuronal apoptosis via upregulation of ERs.	Meng et al. [121]
A preclinical study	Statins may improve gut–brain axis followed by decrease in cell death and amyloid plaques via an anti-inflammatory effect.	Zahedi et al. [122]
A retrospective study	Long-term statin use for >4 years reduces the risk of motor progression.	Palermo et al. [123]
An explorative study	Hydrophilic statins significantly increase clinical and imaging progression in patients with neurodegenerative diseases.	Mechelle et al. [124]
A clinical trial study	Treatment of mild AD/MCI subjects with statins was well tolerated and associated with improvements in CBF and cognitive markers.	Degrush et al. [125]
A clinical trial study	High-intensity statins benefit primary prevention of dementia and MCI in adults older than 75 years.	Philip et al. [126]
A clinical trial study	Statins can protect the microstructure and volume of brain white matter in middle-aged adults.	Vogt et al. [111]
A clinical trial study	Low-dose statins reduce cerebral white matter hyperintensities progression and cognitive decline in hypertensive patients.	Zhang et al. [127]
A systematic review and meta-analysis	Using statins has implications for the treatment of neurodegenerative diseases.	Yan et al. [128]
A systematic review and meta-analysis	Statins may affect plasma Aβ transport and especially increase Aβ42 levels in patients with hyperlipidemia.	Shan et al. [129]
A systematic review and meta-analysis	Short-term statins treatment may beneficially modify CSF biomarkers of AD.	Li et al. [130]
A systematic review and meta-analysis	Statins use has a protective role against the risk of neurodegenerative disease.	Wu et al. [131]

## Abbreviations

ABCA1: ATP-binding cassette transporter A1; AD: Alzheimer’s disease; Aβ: amyloid beta; APOE: apolipoprotein E; APP: amyloid precursor protein; BBB: blood–brain barrier; BDNF: brain-derived neurotrophic factor; COX: cyclooxygenase; CYP46A1: cytochrome P450 46A1; CNS: central nervous system; CRE: cyclic AMP response element; FPP: formation of acetylene pyrophosphate; ER: endoplasmic reticulum; eNOS: endothelial nitric oxide synthase; ERE: estrogen response element; GGPP: geranylgeranyl pyrophosphate; HFD: high-fat diet; HMGCR: 3-hydroxyl 3-methylglutaryl CoA reductase; iNOS: inducible nitric oxide synthase; INSIG: insulin-inducible genes; IPP: isopentenyl pyrophosphate;LDL: low-density lipoprotein; LDL-C: low-density lipoprotein cholesterol; LDLR: low-density lipoprotein cholesterol receptor; IL-1: Interleukin-1; IL-1β: Interleukin-1β; IL-6: Interleukin-6; IR: insulin resistance; MBTPS1: membrane-bound transcription factor site-1 protease; MCI: mild cognitive impairment; MCP-1: monocyte chemoattracting protein-1; MVA: mevalonate; NC: normal cognitive state; NOXs: NADPH oxidases; NF-κB: nuclear factor kappa B; PVN: paraventricular nucleus; RNS: reactive nitrogen species; ROS: reactive oxygen species; SCAP: SREBP cleavage activator protein; SRE: sterol regulatory element; SREBP-2: sterol regulatory element binding protein-2; TNF-α: tumor necrosis factor; 24S-OHC: 24-hydroxy cholesterol; 27-HC: 27-hydroxy cholesterol.
